# BMC Hematology reviewer acknowledgement 2014

**DOI:** 10.1186/s12878-015-0021-9

**Published:** 2015-02-25

**Authors:** Nawsheen Boodhun

**Affiliations:** BioMed Central, Floor 6, 236 Gray’s Inn Road, London, WC1X 8HB UK

## Abstract

The editors of *BMC Hematology* would like to thank all our reviewers who have contributed to the journal in Volume 14 (2014).

**Salem Abbes**

Tunisia

**Omar Aljitawi**

USA

**Tanu Anand**

India

**Victoria Arija**

Spain

**Aranya Bagchi**

USA

**Samir K. Ballas**

USA

**Andrew Blann**

UK

**Kyle Bradley**

USA

**Carol Briggs**

UK

**Jo Caers**

Belgium

**Elena Cassinerio**

Italy

**Daniel Catovsky**

UK

**Harish Chandra**

India

**Ram K Chandyo**

Nepal

**Ying-Jun Chang**

China

**Annalisa Chiappella**

Italy

**Claudio Cimminiello**

Italy

**William Clark**

Canada

**Michael Clemente**

USA

**Philippe Connes**

Guadeloupe

**Alessandro Corso**

Italy

**Sharon Cox**

UK

**Anna Czyz**

Poland

**Wim De Kort**

Netherlands

**Masoud Dehdashtian**

Iran

**Enrico Derenzini**

Italy

**Ahmet Dogan**

USA

**Marco Paolo Donadini**

Italy

**Mehmet Ali Erkurt**

Turkey

**Lucia Farina**

Italy

**Jonathan Flanagan**

USA

**Ivan Gentile**

Italy

**Sophie Georgin-Lavialle**

France

**Thelma T Goncalez**

USA

**Pankaj Gupta**

USA

**Hans Erik Heier**

Norway

**Prashant Hiwarkar**

UK

**Alessandro Jenkner**

Italy

**Andrew Kerkhoff**

USA

**Michael Linden**

USA

**Chen Liu**

USA

**Elena Lukina**

Russian Federation

**Cuzzola Maria**

Italy

**Ismail Matalka**

Jordan

**Juan Mayordomo-Colunga**

Spain

**Saurabh Mehta**

USA

**Amha Mekasha**

Ethiopia

**Erin Meyer**

USA

**Gregorio Paolo Milani**

Italy

**Caterina Minniti**

USA

**Leonard Minuk**

Canada

**Daya Moodley**

South Africa

**Herman Nilsson-Ehle**

Sweden

**Horatiu Olteanu**

USA

**Sjoukje Oosting**

Netherlands

**William Kwame Boakye Ansah Owiredu**

Ghana

**Laura Palareti**

Italy

**Vincent Pialoux**

France

**Lisa Pieri**

Italy

**Kishor Raja**

UK

**Suely Rezende**

Brazil

**Vincent Ribrag**

France

**Paola Saracco**

Italy

**Marie P Schneider**

Switzerland

**Marie Scully**

UK

**David Snyder**

USA

**Anthony Staines**

Ireland

**Virginia Stallings**

USA

**Junko Tanaka**

Japan

**Kickler Tom**

USA

**Ankica Vasilj**

Croatia

**Lilly M Verhagen**

Netherlands

**Anders Vik**

Norway

**Jan Voorberg**

Netherlands

**Milena Vuica-Ross**

USA

**Dan Waxman**

USA

**Ambroise Wonkam**

South Africa

**Abdulraheem Yacoub**

USA

**C. Cameron Yin**

USA

**Binglan Yu**

USA

